# Simulation of Land Use Pattern Evolution from a Multi-Scenario Perspective: A Case Study of Suzhou City in Anhui Province, China

**DOI:** 10.3390/ijerph18030921

**Published:** 2021-01-21

**Authors:** Rongtian Zhang, Jianfei Lu

**Affiliations:** New Rural Development Research Institute, Yangzhou University, Yangzhou 225009, China; jianfeilu@yzu.edu.cn

**Keywords:** land use, landscape pattern, CLUE-S, spatial–temporal simulation, many situations, Suzhou

## Abstract

Land use/land cover change is a frontier issue in the field of geography research. Taking Suzhou City in Anhui Province as the research case, based on thematic mapper /enhanced thematic mapper+ (TM/ETM+) remote sensing data from 1998 to 2018, through the transfer matrix model and modified conversion of land use and its effects at small region extent (CLUE-S) model, the simulation of the land use landscape pattern evolution was studied from a multi-scenario perspective. The results showed that in the past 20 years, landscape patterns have undergone spatial–temporal conversion, which was mainly manifested as the evolution from a cultivated land landscape and other agricultural land to construction land, and there was some transformation between other landscape types, but the transformation degree was not significant. The spatial autocorrelation factor was introduced to correct the CLUE-S model, and the Kappa index reached 0.83, indicating that the modified CLUE-S model had a good simulation accuracy. (I) In the cultivated land protection scenario, limiting the conversion of basic farmland use, and by 2028, the proportion of cultivated land increased by 5.23%, distributed in eastern Suzhou City; (II) in the economic development scenario, by 2028, the construction land area increased by 14.58%, and was distributed in the surrounding regions of the built-up areas; and (III) in the ecological protection scenario, by 2028, wood land, water, and other ecological protection land area increased, and were distributed in the central and eastern part of Suzhou City. Research can provide useful decision-making support for land use optimization and remediation.

## 1. Introduction

Land use/land cover change (LUCC) is a frontier issue in the field of geography research [1]. In recent years, 3S technology, landscape ecology, and other methods for the study of regional land use landscape have increasingly become an important discussion topic. Carrying out an LUCC simulation analysis is helpful to understand the process of land use/land cover change, as well as the impact of various human decisions on land use/land cover change, and for making a scientific scenario prediction for the future LUCC [2]. Land use/land cover change (LUCC) research focuses on the current situation pattern [3], evolutionary characteristics [4], influence factors [5], trend simulation [6], and so on. The research scale of LUCC has gradually shifted from the large macro-scale (province area [7], river basin [8], etc.) to the medium and small scale (city area [9], county area [10], etc.), and it shows a trend of deepening the scale of discussion.

At present, LUCC models are represented by cellular automata (CA) [11], system dynamics (SD) [12,13], the agent-based model [14,15], and the conversion of land use and its effects at small region extent (CLUE-S) model [16,17], etc. The CLUE-S model was first created on the basis of the CLUE model by Verburg of Wageningen University in the Netherlands [18]. The CLUE-S model has a significant advantage by simulating the competitive transfer relationship among various land use landscape types. It can simulate and predict the land landscape pattern under different scenarios, which can help to realize the spatial expression of the future land use landscape pattern [19,20]. Currently, the CLUE-S model is widely used in land use/land cover change [21,22,23], urban spatial expansion [24,25], and other related research fields. In China, research on the CLUE-S model started relatively late, and focused on empirical research with different spatial scales using the CLUE-S model [26,27]; at the same time, other scholars have optimized and modified the CLUE-S model [28,29]. The CLUE-S model can effectively reflect the trend of land use landscape patterns by integrating the evolution process of different spatial–temporal scales and scenario schemes. However, the CLUE-S model still has some deficiencies, which are mainly reflected by the fact that it ignores the influence of spatial autocorrelation on the interaction between various variables, and spatial self-correlation analysis can simulate the dynamic evolution of land use landscape patterns more reasonably. Therefore, in the traditional CLUE-S model, spatial autocorrelation impact factors were introduced in the form of “space rights”, so as to modify the CLUE-S model and to more scientifically simulate the evolution process of regional land use landscape patterns.

Therefore, from the perspective of a multi-scenario study, this paper used the modified CLUE-S model to study the evolution of a typical regional land use pattern. This paper mainly included two parts, namely: (1) What changes have taken place in land use patterns? Based on a thematic mapper/enhanced thematic mapper+ (TM/ETM+) remote sensing image of Suzhou city from 1998 to 2018 as the data source, the land use landscape types of Suzhou were interpreted, and the historical evolution characteristics of the land use landscape pattern were analyzed using the transfer matrix model. (2) How do land use patterns evolve under different scenarios? Under three different scenarios (cultivated land protection, economic development, and ecological protection), the evolution trend of the land use pattern was analyzed, and the optimization methods of the land use pattern under different policy guidance strategies were reflected.

## 2. Materials and Methods

### 2.1. Study Area

**Figure 1:**
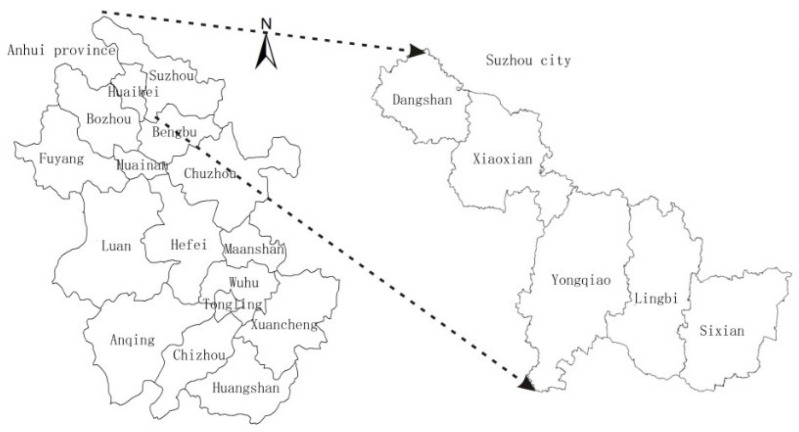
The geographical location of Suzhou City.

Suzhou City is located in the northern part of Anhui Province, between east longitude 116°09′~18°10′, north latitude 33°18′~34°38′, at the junction of the Jiangsu, Henan, Anhui, and Shandong provinces (Figure 1). The land area of Suzhou City is 9939.80 square kilometers, taking up 7.1% of the province’s land area. There is one district, namely Yongqiao District, and four counties, namely, Sixian County, Lingbi County, Xiaoxian County, and Dangshan County. By the end of 2018, the GDP of Suzhou reached 1630.22 million yuan, and the per capita GDP reached 28,757 yuan, compared with southern Jiangsu (per capita GDP of 160,747 yuan). On the whole, Suzhou belongs to a typical city of less developed areas in central China. Through the case study of Suzhou in Anhui Province, this paper aims to provide a case reference for the study of land use landscape patterns in less developed areas of China.

### 2.2. Data Collection

The research data mainly include two parts: (1) TM/ETM+ remote sensing images, which were data from the US Landsat satellite. The imaging time was three time sections on 26 October 1998, 28 October 2008, and 25 October 2018. The resolution was 30 m × 30 m. (2) The economic statistics were all from the Anhui Statistical Yearbook, Suzhou Statistical Yearbook, and the Suzhou National Economic and Social Development Statistical Bulletin (1999, 2009, and 2019).

### 2.3. Research Methods

#### 2.3.1. Transfer Matrix Model

The transfer matrix model does not only show the change in the land use landscape and the flow direction between landscape types, but also quantitatively explains the transformation characteristics between the land use landscape types, which can effectively reveal the spatial–temporal evolution rules of the regional land use landscape pattern. The basic principle of the transfer matrix model is that according to the map algebra and its Markova process principle, the land use landscape map in any two periods, $A_{i\times j}^{T}$ and $A_{i\times j}^{T+1}$, are obtained by analyzing the map algebra operation, using the arithmetic of map algebra, $C_{i\times j}=A_{i\times j}^{T}\times 10+A_{i\times j}^{T+1}$. In this paper, the matrix function in the interpreter module of the ERDAS software was used to calculate the transfer diagram of the land use landscape types in each time period, as well as to organize the transfer matrix of the land use landscape type in the research area [30,31,32].

#### 2.3.2. The Modified CLUE-S Model

The traditional CLUE-S model ignores the effects of spatial self-correlation, trying to introduce the influence factor of spatial auto-correlation in the form of “space right” in the traditional logic model. We then constructed an auto logistic analysis model, which could effectively solve the spatial self-correlation effect inherent in the simulation [33,34,35].

If using the set {(*X_i_*, *T_i_*), *i* = 1, 2 …, n} indicates the spatial location and occurrence time of all the land use landscape types, and the conditional probability of a certain land use landscape type appearing in spatial–temporal location (*X_i_*, *T_i_*) satisfies the basic form of the logistic function, then the conditional probability of the land use landscape type appearing can be expressed as the following theoretical form:

(1) $$P=\left(y_{i}=1|\beta_{0},\beta ,r\right)=\exp\left(\beta_{0}+\beta^{\prime }X_{i}+r\sum W_{i}{}_{j}\right)/1+\exp\left(\beta_{0}+\beta^{\prime }X_{i}+r\sum W_{i}{}_{j}\right)$$

where *W_ij_* is the spatial weight function of points *i* and *j*, and *W_ij_* is the reciprocal of the distance between the point pairs. In other words, if the distance between the spatial points to *i* and *j* is represented by *D_ij_*, the *W_ij_* is calculated as follows:

(2) $$W_{ij}=\left\{ \begin{array}{l} 1/D_{ij}\\ 0 \end{array}\right.$$

**Figure 2:**
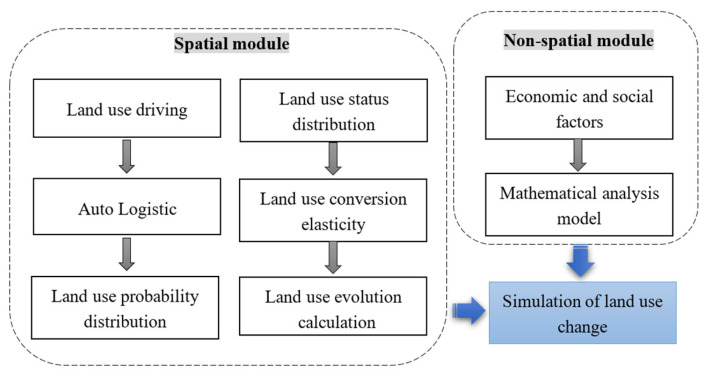
Schematic diagram of the modified CLUE-S mode.

The CLUE-S model mainly includes a spatial module and non-spatial module (Figure 2) [36]. ① Spatial module is based on the raster spatial database, using the adaptive probability distribution of the raster land use type, the stability of various land use landscape types, and the land use landscape conversion rules, and the change in the region land demand scale is allocated to each candidate unit. ② The non-spatial module calculates the annual scale change of the land use demand in the research area, by analyzing the driving factors of the land use landscape change, such as population, economy, society, policies, and regulations. The specific operating principle of the modified CLUE-S model is shown in Figure 2.

## 3. Results

### 3.1. Spatial–Temporal Evolution of Land Use Patterns

**Figure 3:**
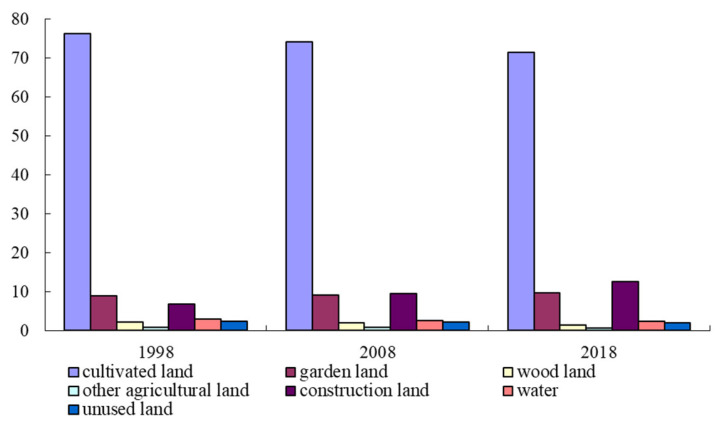
The proportion of land use type distribution from 1998 to 2018.

The spatial–temporal evolution of land use patterns was based on ERDAS9.1 software (Erdas, Atlanta, GA, USA), according to TM/ETM^+^ remote sensing imagery from 1998 to 2018. Considering the actual situation of land use in Suzhou City, combined with the preparation of the general plan for land use at the municipal level (TD/T 1023-2010), using a supervisory classification, the Suzhou City land use types were divided into cultivated land, garden land, wood land, other agricultural land, construction land, water, and unused land. Based on the ArcGIS10.2 software platform (Environmental Systems Research Institute, Redlands, USA), the data information characteristics of the area and proportion of land use landscape types in the third phase of 1998, 2008, and 2018 in Suzhou City were counted (Figure 3).

Through the proportion distribution of land-use landscape types in Suzhou City from 1998 to 2018, it can be seen that the proportion of cultivated land landscape was the largest (70.37, 76.23), this was followed by the land use type of garden (8.88, 9.65) and construction land (6.76, 12.49), while the proportion of other agricultural land (0.65, 0.81) and unused land (2.05, 2.32) were relatively small. However, the characteristics of land use landscape types in Suzhou city from 1998 to 2018 need to be explored further. In view of this, this paper used the transfer matrix model to make a preliminary analysis of the evolution process between land use landscape types in Suzhou City from 1998 to 2018, and tried to reveal the internal transformation relationship between land use landscape types in the region (Table 1 and Table 2).

It can be seen that (1) the change of cultivated land landscape to construction land landscape was a relatively significant feature of the land use landscape transformation in Suzhou City. Here, 4.56% of cultivated land landscape was converted to construction land from 1998 to 2008, and 5.65% of cultivated land landscape was converted to construction land from 2008 to 2018, showing a trend of increasing conversion rate. (2) Wood land was mainly converted into garden land, followed by cultivated land. From 1998 to 2008, the conversion rate reached 0.59% and 0.87%; from 2008 to 2018, the conversion rate reached 0.43% and 0.56%. (3) During the research period, the main transfer direction of garden land was construction land, and the conversion rates were 1.45% and 1.56%. (4) The most important transfer direction of water and unused land was cultivated land. From 1998 to 2008, the conversion rates were 0.51% and 0.12%; from 2008 to 2018, the conversion rates were 0.42% and 0.08%. Water and unused landscape were relatively stable during the study period. (5) On the whole, there was a certain transformation relationship among all land use landscape types in Suzhou City from 1998 to 2018, but the transformation of cultivated land landscape to construction land was the most prominent, which was mainly attributed to the response of human activities such as urban construction and agricultural development.

### 3.2. Multi-Scenario Simulation of Land Use Pattern

#### 3.2.1. Logistic Regression Analysis of Driving Factors

According to the actual regional characteristics of Suzhou City, this paper selected ten driving factors, namely, distance from cities and towns, distance from rural areas, distance from railways, distance from highways, distance from rivers, elevation, slope, slope direction, population density, and urbanization rate. Based on the SPSS17.0 (International Business Machines Corporation, Armonk, NY, USA) software platform, a binary logistic module was used to carry out the regression analysis on various land use landscape types and their driving factors in Suzhou City at the spatial scale of 300 m × 300 m. (1) We converted the ASCII code files of seven types of land use landscape and ten driving factors of cultivated land, garden land, wood land, other agricultural land, construction land, water and unused land into a single file format through file converter. (2) The results were input into SPSS17.0 software, and the seven land use landscape types and ten driving factors were analyzed to obtain the regression models and corresponding regression parameters of various land use types in Suzhou City (Table 3).

#### 3.2.2. ROC Validity Test

The relative operating characteristic (ROC) is a method to verify the effectiveness of the auto logistic regression model. If the ROC value is between 0.5 and 1, and the closer the ROC value is to 1, it has been shown that the probability distribution of a geoid is more consistent with the spatial distribution of a real geoid, and the better the regression equation can explain the spatial distribution. On the contrary, the closer the ROC value is to 0.5, it has been shown that the explanatory significance of the regression equation to the terrestrial spatial distribution is lower. Theoretically, when ROC > 0.7, the selected driving factors have a good explanatory ability. Based on SPSS17.0 software (International Business Machines Corporation, Armonk, NY, USA), ROC values of various land use landscape types in Suzhou City were calculated, the results showed the following: (1) the ROC curves of each class were greater than 0.7, which indicated that the auto logistic model was relatively high. (2) Considering the spatial autocorrelation, the fitting effect of the auto logistic regression model was more improved, and the fitting degree of various land landscape types was 0.826 for cultivated land, 0.851 for garden land, 0.894 for wood land, 0.882 for other agricultural land, 0.843 for construction land, 0.903 for water, and 0.912 for unused land. In general, the auto logistic regression model with spatial autocorrelation was more reasonable in simulating the evolution trend of the land use landscape pattern.

#### 3.2.3. Transform Elasticity and Its Transfer Matrix

**Figure 4:**
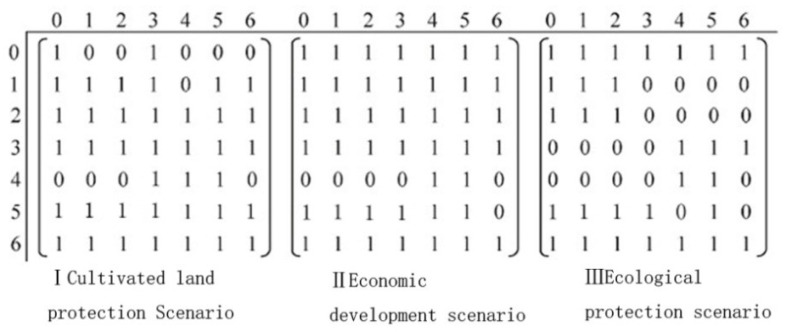
Matrix of land use conversion rules under three scenarios. Out matrix: (0) cultivated land, (1) garden land, (2) wood land, (3) other agricultural land, (4) construction land, (5) water, and (6) unused land. In matrix: (0) not transferable and (1) transferable.

According to the evolution characteristics of Suzhou’s land use landscape pattern, in the future scenario prediction process, the relative elasticity coefficient (*ELAS*) of Suzhou City was set for three scenarios. ① (Ⅰ) The cultivated land protection scenario focused on improving the relative elasticity coefficient of the cultivated land landscape. ② (Ⅱ) The economic development scenario reduced the *ELAS* value of the cultivated land, other agricultural land, and other landscape. ③ (Ⅲ) The ecological protection scenario increased the *ELAS* value of the wood land, garden land, unused land, water area, and other landscape (Table 4). Meanwhile, the CLUE-S model needed to determine the transfer matrix between land use landscape types; based on the transfer characteristics of the land use landscape types in Suzhou City from 1998 to 2018, the transfer matrix of the land use landscape types in Suzhou City was set for three scenarios (Figure 4).

#### 3.2.4. Land Use Pattern Scenario Simulation

First of all, the modified CLUE-S model was used to simulate the land use landscape pattern distribution in Suzhou City in 2018; based on ERDAS9.1 software (Erdas, Atlanta, GA, USA), the accuracy assessment was used for testing, and the Kappa index was 0.83. Theoretically, if Kappa ≥ 0.75, the landscape distribution of the two land uses had a relatively high consistency, which indicated that the modified CLUE-S model has a high simulation accuracy.

Secondly, according to the characteristics of the land use landscape pattern in Suzhou City, three basic scenario scenarios were set (cultivated land protection scenario, economic development scenario, and ecological protection scenario). The scale of the land use landscape type was scientifically estimated using a Markov model and (1,1) gray model (GM), separately (Table 5).

Finally, the demand scale of various land use landscape types in different scenarios in 2028 was brought into the CLUE-S model as an important parameter; based on the current situation of land use in Suzhou City in 2018, the evolution trends of the land use landscape Bureau in Suzhou City under three different scenarios in the next ten years (2018–2028) were simulated.

**Figure 5:**
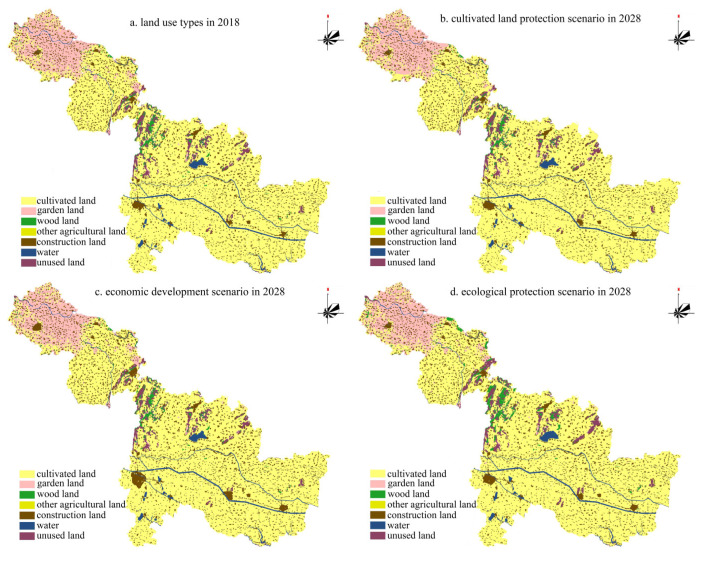
Land use map in 2018 and simulated results in 2028 under different scenarios.

(1) (Ⅰ) The cultivated land protection scenario. The change in the nature of the cultivated land landscape type use in the area of Suzhou City is controlled, and the spatial distribution of the cultivated land landscape is relatively concentrated, and is mainly distributed in Sixian County, Xiaoxian County, and Yongqiao District. By 2028, the area of cultivated land will increase by 5.23%. The decreased cultivated land is mainly distributed in Dangshan County; this region is short of water resources and has a high terrain, which is suitable for planting gardens. In this scenario, by restricting the rapid growth of the construction land landscape type, the overall construction land landscape of Suzhou City showed a slight increase, mainly distributed in the surrounding areas of the built-up area, while the water landscape type distribution was relatively stable.

(2) (Ⅱ) The economic development scenario. Compared with the other two scenarios, the stability of construction land and cultivated land is the worst, the prominent performance is that if the scale of regional construction land shows a significant increase, cultivated land and other agricultural land types decreases sharply. By 2028, the construction land area of Suzhou will increase by 14.58%; for the spatial distribution, the significant expansion area of construction land is mainly concentrated in the southeast of Yongqiao District, especially the Bianhe New District and the economic development zones. In addition, Xiaoxian County, Sixian County, Dangshan County, and Lingbi County are also expanding on a certain scale.

(3) (Ⅲ) The ecological protection scenario. The landscape types of wood land, water body, and unused land are well protected, and the expansion of the urban and rural construction land area is controlled. By 2028, the proportion of wood land will increase by 3.89%, which is mainly distributed in central Suzhou, and represents the distribution characteristics of “concentrated film”. By 2028, water land types will also increase by 2.02%, mainly distributed in Lingbi County and Sixian County in eastern Suzhou. In addition, the spatial distribution of cultural heritage protected areas is stable in Suzhou. (Figure 5).

**Figure 6:**
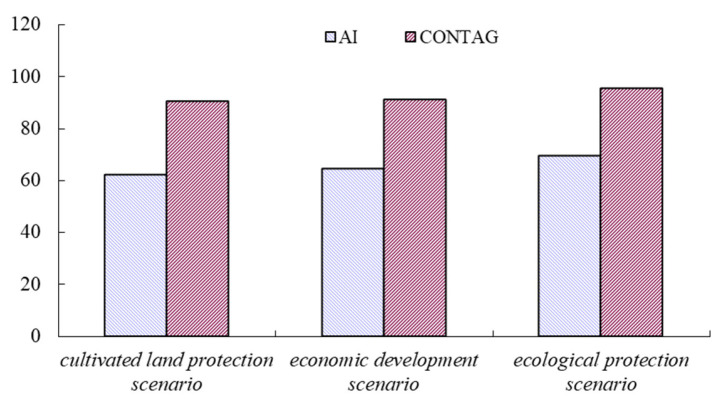
Comparison of landscape index under three scenarios in Suzhou.

In order to compare the simulation results of the land use landscape pattern evolution under three scenarios in Suzhou City, the degree of polymerization (AI) and degree of spread (CONTAG) were selected to analyze the different characteristics of the land use landscape pattern in Suzhou City (Figure 6). The following is presented in Figure 6: ① the AI and CONTAG of the land use landscape pattern under the ecological protection scenario are the largest, which shows that the spatial aggregation of the land use landscape pattern under the scenario is the highest and its connectivity is the best. ② The spatial heterogeneity of the land use landscape pattern under the cultivated land protection scenario and economic development scenario are strong, indicating that the spatial distribution is relatively scattered. ③ In addition, collectively, (Ⅰ) cultivated protection scenario, (Ⅱ) economic development scenario, and (Ⅲ) ecological protection scenario show that there is a high similarity between the simulated regional land use pattern spatial evolution under the economic development scenarios and the general land use planning map of Suzhou; it shows that the future of Suzhou City is in the accelerated period of urbanization development. Therefore, it is necessary to make reasonable and scientific planning for future land use in Suzhou City, not only to promote regional economic development, but also to ensure sustainable land use, which is the focus of the land resource management policy in Suzhou City for the future.

## 4. Discussion

(1) The spatial autocorrelation factor was introduced to modify the CLUE-S model, and the Kappa index value reached 0.83 at the 300 m × 300 m scale, which showed that the modified CLUE-S model has a better simulation accuracy. Meanwhile, for different scales (100 m × 100 m, 200 m × 200 m, 500 m × 500 m, and 800 m × 800 m), what is the simulation accuracy of the modified CLUE-S model? A comparative study on different spatial scales will be an important direction to investigate. In addition, the modified CLUE-S model is not limited to a city, but is also applicable to a larger scale area.

(2) The modified CLUE-S model is more reasonable for simulating land use demand and spatial allocation, but selection of the driving factors is objective and quantifiable, and policy factors have an important influence on the evolution of the regional land use pattern. At present, it is difficult to quantify the influence of policy factors. How will one be able to quantify the driving effect of the policy factors? This will be the research direction of the land use landscape pattern simulation in the future.

## 5. Conclusions

(1) The land use landscape pattern has undergone spatial–temporal transformation in Suzhou since 1998. It was mainly manifested in the landscape evolution from cultivated land and other agricultural land to construction land. The change from the cultivated land landscape to construction land was the most significant feature in Suzhou. From 1998 to 2008, 4.56% of the cultivated land landscape was converted into construction land, and 5.65% of the cultivated land landscape was converted into construction land, showing an increasing trend with a conversion rate. At the same time, there was a certain transformation among other land use landscape types. Human activities (agricultural cultivation, urban construction, etc.) play an important role in the evolution of the regional land use landscape pattern.

(2) There are different land use landscape pattern evolution characteristics under different scenarios. (Ⅰ) In the cultivated land protection scenario, by limiting the conversion of basic farmland use and restricting the rapid expansion of construction land, by 2028, the proportion of cultivated land landscape types will increase by 5.23%, distributed in the east of Suzhou. (Ⅱ) In the economic development scenario, construction land space is rapidly expanded, and by 2028, the area will increase by 14.58%, which mainly encroaches on a large amount of cultivated land around urban areas. (Ⅲ) In the ecological protection scenario, by 2028, the area of wood land, water, and other ecological protection land will increase and be distributed in eastern Suzhou City. The scenario simulation scheme has a practical reference value for the adjustment of regional land use planning.

## Figures and Tables

**Table 1:** Transformation matrix of landscape types in Suzhou City from 1998 to 2008 (%).

1998	2008						
	Cultivated Land	Garden Land	Wood Land	Other Agricultural Land	Construction Land	Water	Unused Land
Cultivated land	93.14	1.23	0.02	1.02	4.56	0.03	0.00
Garden land	0.13	98.27	0.59	0.12	1.45	0.12	0.01
Wood land	0.43	0.03	98.14	0.23	0.35	0.13	0.00
Other agricultural land	0.45	0.87	0.13	95.75	2.56	0.23	0.01
Construction land	0.43	0.23	0.12	0.13	99.07	0.02	0.00
Water	0.51	0.36	0.23	0.55	0.49	97.84	0.02
Unused land	0.12	0.16	0.11	0.11	0.18	0.11	99.21

**Table 2:** Transformation matrix of landscape types in Suzhou City from 2008 to 2018 (%).

2008	2018						
	Cultivated Land	Garden Land	Wood Land	Other Agricultural Land	Construction Land	Water	Unused Land
Cultivated land	91.55	1.54	0.09	1.12	5.65	0.05	0.00
Garden land	0.26	97.48	0.87	0.23	1.56	0.23	0.03
Wood land	0.56	0.13	97.56	0.43	0.55	0.11	0.00
Other agricultural land	0.56	0.79	0.32	95.08	2.89	0.35	0.01
Construction land	0.35	0.22	0.11	0.16	99.15	0.01	0.00
Water	0.42	0.36	0.25	0.42	0.45	98.09	0.01
Unused land	0.08	0.14	0.12	0.07	0.12	0.07	99.4

**Table 3:** The results of the auto logistic regression for different land use types.

Driving Factor	Cultivated Land		Garden Land		Wood Land		Other Agricultural Land		Construction Land		Water		Unused Land	
	Beta	Exp (β)	Beta	Exp (β)	Beta	Exp (β)	Beta	Exp (β)	Beta	Exp (β)	Beta	Exp (β)	Beta	Exp (β)
Constant quantity	0.453	1.563	0.165	1.003	−0.187	0.894	0.078	0.956	0.198	0.967	0.289	0.987	−0.023	0.903
Distance from cities	0.019	0.865	−1.098	0.799	−0.035	0.797	0.021	0.876	0.082	1.076	0.002	0.708	−0.089	0.773
Distance from rural	0.042	1.087	0.024	0.902	-	-	0.045	0.892	0.024	0.878	0.023	0.854	0.045	0.928
Distance from railways	-	-	-	-	-	-	-	-	0.012	0.862	-	-	-	-
Distance from highways	-	-	-	-	-	-	0.004	0.862	0.045	0.893	-	-	-	-
Distance from rivers	-	-	0.021	0.898	0.063	1.002	0.089	0.895	0.067	0.954	0.073	1.045	0.043	0.913
Elevation	−0.028	0.776	0.026	0.921	0.012	0.834	0.056	1.034	0.057	0.923	0.047	0.892	0.036	0.897
Slope	−0.078	0.788	0.058	1.024	0.067	0.949	0.026	0.784	0.035	0.883	0.054	0.895	0.034	0.894
Slope direction	−0.062	0.767	0.067	1.035	0.076	1.028	0.064	1.045	0.038	0.886	0.068	1.003	0.056	1.005
Population density	0.042	1.043	−0.754	0.796	0.032	0.894	0.082	1.056	0.074	1.056	0.089	1.045	0.002	0.793
Urbanization rate	0.028	1.008	0.076	1.047	0.034	0.903	0.076	1.034	0.087	1.082	0.087	1.041	−0.034	0.703
Auto value	0.001	1.004	0.001	1.002	0.002	1.005	0.001	1.002	0.002	1.006	0.004	0.713	0.002	1.001
ROC	0.826		0.851		0.894		0.882		0.843		0.903		0.912	

**Table 4:** The *ELAS* of land use landscape types under different scenarios.

Land Use Type	Ⅰ Cultivated Land Protection Scenario	Ⅱ Economic Development Scenario	Ⅲ Ecological Protection Scenario
Cultivated land	0.9	0.7	0.9
Garden land	0.8	0.8	0.8
Wood land	0.7	0.9	0.8
Other agricultural land	0.8	0.8	0.7
Construction land	0.9	0.9	0.9
Water	0.8	0.8	0.9
Unused land	0.9	0.9	0.9

**Table 5:** The land use demand in three scenarios of Suzhou in 2028 (hm^2^).

There Scenarios	Cultivated Land	Garden Land	Wood Land	Other Agricultural Land	Construction Land	Water	Unused Land
(Ⅰ) cultivated land protection scenario	709,615.91	95,922.76	13,411.77	24,510.55	104,321.36	23,207.47	27,370.28
(Ⅱ) economic development scenario	702,089.75	95,758.12	12,776.65	21,694.85	119,964.78	21,988.85	24,087.07
(Ⅲ) ecological protection scenario	709,608.49	96,013.18	13,557.81	22,354.75	104,539.08	24,228.23	28,058.55
